# POU5F1/Oct-4 expression in breast cancer tissue is significantly associated with non-sentinel lymph node metastasis

**DOI:** 10.1186/s12885-015-1966-6

**Published:** 2016-03-01

**Authors:** Shouliang Cai, Shugang Geng, Feng Jin, Jisheng Liu, Chang Qu, Bo Chen

**Affiliations:** Department of Breast Surgery, The First Hospital of China Medical University, #155, Nanjingbei Street, Heping District, Shenyang, Liaoning Province PR China; Department of Surgery, Ansteel Group Hospital, No.3, Jianshen Road, Tiedong District, Anshan, Liaoning Province PR China

**Keywords:** Breast cancer, SLN, Non-SLN, Octamer binding factor, MSKCC nomogram

## Abstract

**Background:**

At present, few studies have explored the significance of POU5F1 (also known as octamer-bingding factor, Oct-4 or Oct-3) expression in breast cancer tissues.

**Methods:**

A total of 121 patients were retrospectively selected between May 2010 and March 2013 to investigate the relationship between POU5F1/Oct-4 expression in breast cancer tissues and non-sentinel lymph node (non-SLN) metastases and to validate the Memorial Sloan-Kettering Cancer Center (MSKCC) nomogram. All patients had early-stage breast cancer, which was histologically confirmed by the Department of Surgical Oncology, The First Affiliated Hospital of China Medical University. Histological type and grade of tumors were determined from tissue samples by hematoxylin and eosin staining, while the presence of POU5F1**/**Oct-4 protein was determined by immunohistochemistry. POU5F1/Oct-4 expression levels in tissues obtained from patients with sentinel lymph node (SLN) and non-SLN metastasis and in tissues obtained from patients without lymph node metastases were compared.

**Results:**

POU5F1**/**Oct-4 expression levels in breast cancer tissues were significantly higher in both the SLN metastasis and non-SLN metastasis groups (*P* = 0.003 and *P* = 0.030, respectively). Furthermore, POU5F1/Oct-4 expression was found to be associated to both histological (*P* = 0.01) and molecular type (*P* = 0.03). Thus, our data once again confirms the validity of the MSKCC nomogram. The area under curve (AUC) was 0.919 (95 % CI: 0.869–0.969, *P* < 0.001). The probability of non-SLN metastasis generated from the MSKCC nomgram was significantly higher in the POU5F1**/**Oct-4 positive group than in the POU5F1/Oct-4 negative group. Both univariate and multivariate analysis revealed that Oct-4 expression levels were significantly associated with non-SLN metastases (*P* = 0.030 and *P* = 0.034, respectively).

**Conclusions:**

POU5F1**/**Oct-4 expression levels are significantly associated with non-SLN metastases. Patients with higher probabilities of metastasis generated from the MSKCC nomogram may also have higher POU5F1/Oct-4 expression levels.

## Background

Sentinel lymph node (SLN) biopsy is the current standard axillary approach for patients with clinically node-negative breast cancer [1–4]. A complete axillary dissection is often performed to determine whether non-SLNs are involved and whether tumors have occurred in SLNs. Although this approach has been widely accepted, a randomized prospective trial revealed that survival rates in breast cancer patients who underwent axillary lymph node dissection were not superior to survival rates in breast cancer patients who underwent SLN dissection [5]. Therefore, a complete axillary dissection would not be required for patients with SLN but without non-SLN metastasis, if a new approach that can predict the situation of non-SLNs is available. One booming approach is to establish mathematical models using clinical parameters to evaluate the probability of non-SLN metastasis [6] such as the MSKCC nomogram (http://nomograms.mskcc.org/Breast/BreastAdditionalNonSLNMetastasesPage.aspx). The MSKCC nomogram is a multivariate model designed by the Memorial Sloan-Kettering Cancer Center to predict the possibility of lymph node metastasis. This model was established based on a retrospective analysis of 3786 cases that underwent lymph nodes biopsy. Among these cases, 1548 patients were studied to validate the prediction model, yielding an AUC of 0.754 [7]. This indicates that the MSKCC nomogram has excellent clinical value in predicting lymph node metastases. Although this has been the most widely used model, this model was established based solely on patients from medical centers of the United States [7]; which may result in inaccuracy, considering factors such as ethnic or environment. Therefore, the validation of this model through patient’s of other ethnics or from other countries should be encouraged. Furthermore, models such as the MSKCC nomogram are often beyond clinical requirements, even though these relatively exhibit good performance. Another method is to find a predictor of SLN and non-SLN metastasis. Although few predictors are eligible, some potential predictors have already been found [8, 9].

POU5F1, also known as Oct-3 or Oct-4, is a transcription factor in embryonic stem cells (ESCs) [10]; and is associated with the pluripotency, proliferative potential and self-renewal capacity of ESCs and germ cells [11]. During the early-stages of vertebrate development, POU5F1/Oct-4 act as the most important regulator of pluripotency [12]. Previous studies have shown that POU5F1/Oct-4 plays an extremely important role in the maintenance of the normal stem cell self-renewal process [10, 11, 13, 14]. This stem cell marker, which is significantly reduced in both cytoplasm in vitro and in vivo, differentiated somatic cells [15]. It has also been considered that POU5F1/Oct-4 is associated with cancer, since there are many similarities between ESCs and cancer cells [15]. Several studies have reported the expression of POUF1/Oct-4 in human cancer cells. The study of Tai et al. revealed that POUF1/Oct-4 expression could be found in adult human kidney, breast epithelial, pancreatic, mesenchymal, liver and gastric stem cells [16]. Furthermore, the study of Jin et al. revealed that at least four gene products including Oct-4 were expressed in MCF-7 [17]. Moreover, immunohistochemical examination revealed that POU5F1/Oct-4 expression was significantly elevated in tumor tissues compared to adjacent non-cancerous tissues [18]. Therefore, exploring the association between the expression of POU5F1/Oct-4 in cells and tumors may give insight into novel diagnostic methods.

In the present study, we investigated the relationship of the expression of POU5F1/Oct-4 between breast cancer tissues and non-SLN metastases to determine a substitute for complete axillary dissection during the treatment of breast cancer. Furthermore, this study also aims to validate the MSKCC nomogram in patients from Northeast China, since no published study has validated the MSKCC nomogram in patients from this population in China.

## Methods

### Patients and tissue specimens

Patients histologically diagnosed with early-stage breast cancer by the Department of Surgical Oncology, The First Affiliated Hospital of China Medical University between May 2010 and March 2013 were retrospectively selected for this study. Inclusion criteria were as follows: (I) patients who underwent curative operations with no prior systemic treatment; (II) patients who underwent SLN biopsy and treatment, and patients with positive non-SLN metastases who underwent a complete axillary dissection; (III) patients whose resected specimens were pathologically examined after the operation; and (IV) patients whose complete medical records are available.

### Experimental materials

Polyclonal mouse anti-human Oct-4 antibody (dilution 1:80) was obtained from BIOMOL International. Monoclonal mouse anti-human estrogen receptor (ER) antibody (dilution 1:100), monoclonal mouse anti-human progesterone receptor (PR) antibody (dilution 1:100), monoclonal mouse anti-C-erbB-2 antibody (dilution 1:100), and polyclonal rat anti-human mutant-type tumor protein 53 (P53) antibody (dilution 1:100) were purchased from Zhongshan Golden Bridge Biotechnology Co. Ltd.

### Immunohistochemistry experimental procedures

Thin slices of tumor tissues from all cases received by our histopathology unit were fixed in 4 % formaldehyde solution (pH 7.0) for a maximum period of 24 h. Then, tissues were processed routinely for paraffin embedding; and 4-μm-thick sections were cut and placed onto glass slides coated with (3-aminopropyl) triethoxysilane for immunohistochemistry. Afterwards, tissue samples were stained with hematoxylin and eosin to determine the histological type and grade of the tumors.

Breast tumor tissues obtained from all cases were cut into 4-μm–thick sections using a cryostat. The sections were mounted onto microscope slides, air-dried, and fixed in a mixture of 50 % acetone and 50 % methanol. Then, the sections were dewaxed in xylene, gradually hydrated with gradient alcohol, and washed with PBS. Afterwards, the sections were incubated with 3 % H_2_O_2_for 10 min at room temperature, and incubated overnight at 4°C with the primary antibody. After washing with PBS, the sections were incubated with a polymer helper (PV-9000; Zhongshan Golden Bridge Biotechnology Co, Ltd.) for 20 min at 37°C. Then, after washing with PBS, the secondary antibody (Poly Peroxidase-anti-mouse/rabbit immunoglobulin; Zhongshan Golden Bridge Biotechnology Co, Ltd.) was applied to the sections for 30 min at 37°C. After extensive washings, immunoreactive products were visualized by catalysis of 3, 3-diaminobenzidine (DAB). Then, the sections were counterstained in Gill’s Hematoxylin and dehydrated in ascending grades of methanol before clearing in xylene and mounting under a coverslip.

Oct-4 expression was classified semi-quantitatively according to the following criteria: 0, if <1 % of neoplastic cells discretely expressed POU5F1/Oct-4 in their cytoplasm; 1+, if ≥1 % to <10 % of morphologically unequivocal neoplastic cells discretely expressed POU5F1/Oct-4 in their cytoplasm; 2+, if ≥10 % of morphologically unequivocal neoplastic cells discretely expressed POU5F1/Oct-4 in their cytoplasm. Samples that scored 1+ or 2+ were considered positive.

Nuclear staining for ER, PR and mutant-type P53 were graded as follows: 1+, if <10 % of the cells were stained; 2+, if 10–50 % of the cells were stained; and 3+, if >50 % of the cells were stained. Grades 2+ and 3+ were considered positive, while the absence of staining and grade 1+ were considered negative. Similar standards were used for the staining intensity of HER-2/neu. In this study, only a grade of 3+ (high intensity) was considered positive.

**Fig. 1:**
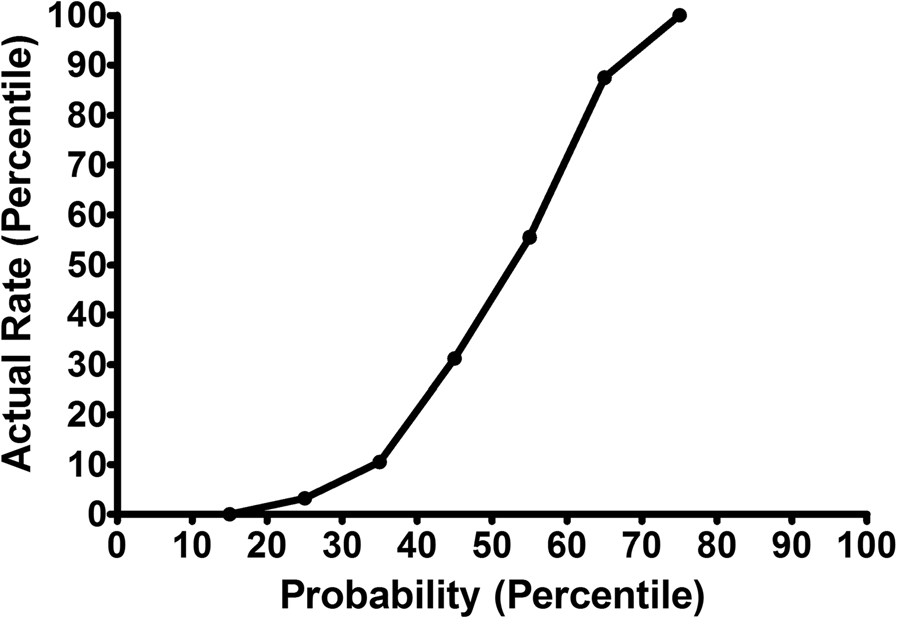
This constructed figure can validate the MSKCC nomogram in predicting SLN metastases. The X-axis represents the probability of SLN metastasis generated by the MSKCC nomogram, and the Y-axis represents the actual SLN metastases rate

In addition, a retrospective review was performed in patients enrolled in this study . All patient data were anonymously used, and only with the sequence number was obtained. Furthermore, pathological diagnosis results were applied to validate the MSKCC nomogram. Figure 1 illustrates the validity of the MSKCC nomogram in predicting SLN metastases. The X-axis represents the probability of SLN metastasis generated by the MSKCC nomogram, while the Y-axis represents the actual SLN metastasis rate. A receiver operating characteristic (ROC) curve was also constructed, and the performance of the established MSKCC model was assessed by calculating the AUC. All procedures in this study were carried out in compliance with the Declaration of Helsinki and were approved by the Ethics Committee of China Medical University (Reference no. 25, 2015), and written informed consents were obtained from all participants.

### Statistical analysis

All statistical analyses were performed using the SPSS statistics software (Version 16.0; SPSS Inc., Chicago, IL, USA). Differences between percentages were calculated using the Chi-square test, and relationships between the expression of POU5F1/Oct-4 and other parameters were explored using the Chi-square test, Fisher’s exact test, or independent *t*-tests. A *P*-value <0.05 was considered statistically significant. All tests were two-sided. Figures were prepared using GraphPad Prism version 5 or SPSS. Univariate analysis was performed to test the prediction of Oct-4 on non-SLN metastases, while multivariate analysis was performed to analyze the expression of Oct-4, ER, PR, Her-2+ and P53 on non-SLN metastases.

## Results

### Patient characteristic

A total of 121 patients were enrolled in this study, and mean age of these patients was 51.9 years (range, 26–81 years). Among these patients, 25 patients had ductal carcinoma *in situ* (DCIS), 85 patients had invasive ductal carcinoma (IDC), nine patients had mucinous carcinoma and two cases had Paget’s disease. None of these patients received prior systemic chemotherapy. Furthermore, among these patients, 83 patients were free from SLN metastasis, 33 patients had pN_1_ metastasis, and five patients had pN_2_ metastasis. Moreover, among all patients included in this study, 52.1 % of patients had tumors that were POU5F1/Oct-4 positive, 53.7 % of patients were ER-positive, 43.0 % of patients were PR-positive, and 91.7 % of patients were HER2/neu-negative. Patient characteristics are listed in detail in Table 1.

**Table 1:** Characteristics of patients included in the study

Variables	Number	Percent (%)
Number of patients	121	
Age		
Oct-4 positive	63	52.1
Size of tumor		
T1 (Tis)	73	60.3
T2	45	37.2
T3	3	2.5
Pathology classification		
DCIS	25	20.7
IDS	85	70.2
Mucinous carcinoma	9	7.4
Paget’s disease	2	1.6
ER positive	65	53.7
PR positive	52	43.0
HER2/neu expression (3+)	10	8.3
Histological grade		
I	20	16.5
II	89	73.6
III	12	9.1
Metastasis		
pN_0_	83	68.6
pN_1_	33	27.3
pN_2_	5	4.1

### Correlation between POU5F1/Oct-4 expression and clinicopathological features

**Fig. 2:**
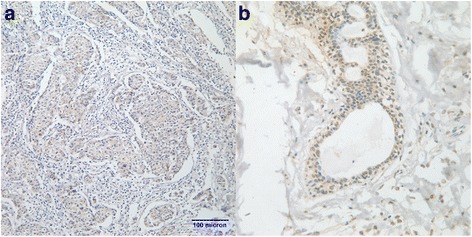
When Oct-4 was overexpressed in the tissue, HER2 was also overexpressed in the same tissue: **a**. magnification × 200; **b**. magnification × 400

**Fig. 3:**
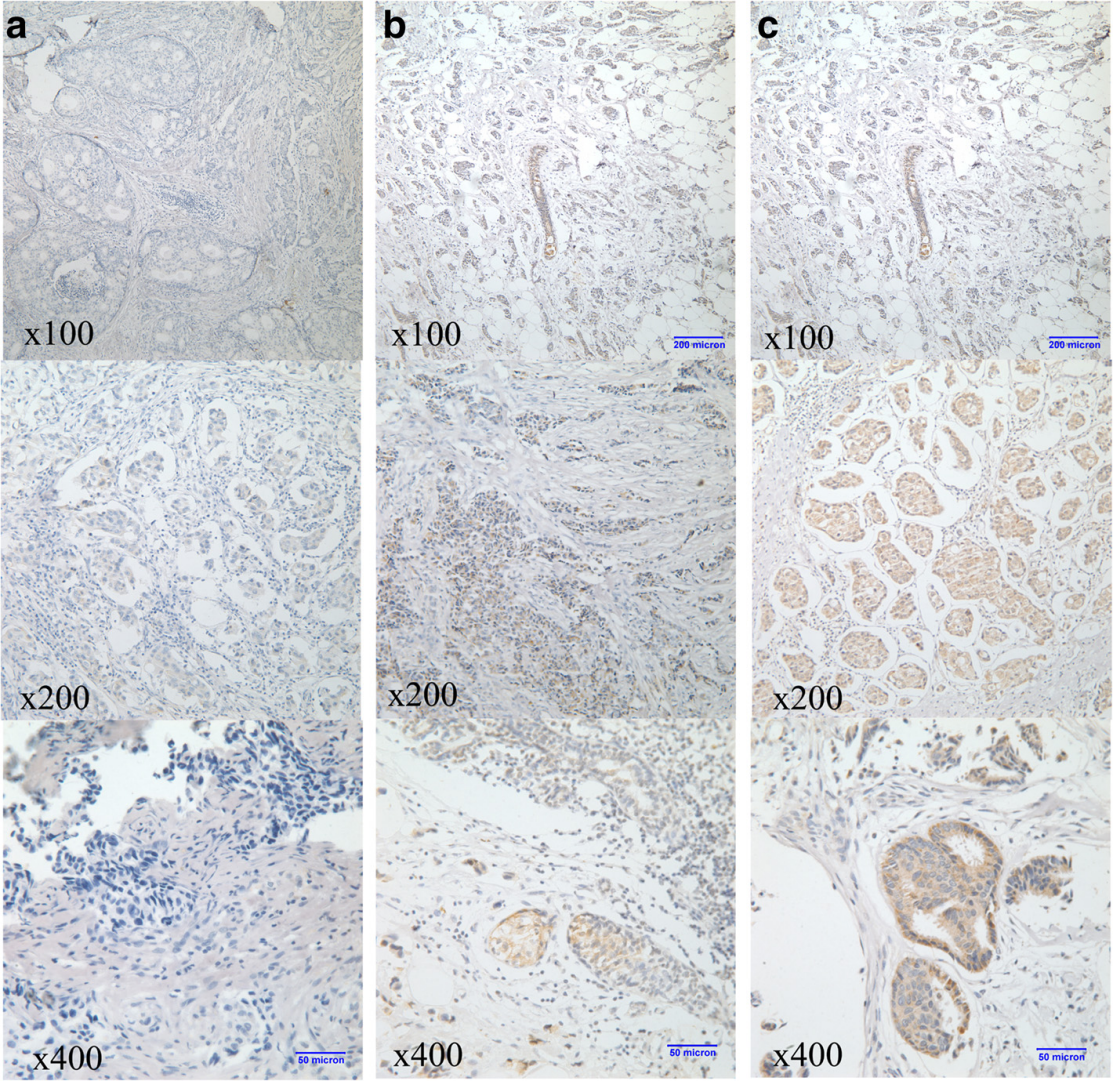
Representative photos of Oct-4/ POU5F1 staining: **a**. negative expression of POU5F1/Oct-4 (score 0); **b**. weak-positive expression of POU5F1/Oct-4 (score 1+); **c**. positive expression of POU5F1/Oct-4 (score 2+)

Overall, the expression of POU5F1/Oct-4 was not associated with age, tumor size or stage. However, it was significantly correlated to histological grade, molecular typing and SLN metastasis. A high proportion of samples with elevated POU5F1/Oct-4 expression levels were observed in patients with SLN metastasis (42.17 % *vs.* 69.70 % *vs.* 100.00 % for pN_0_, pN_1_ and pN_2_, respectively; *P* = 0.003). POU5F1/Oct-4 expression levels were significantly higher in tissues of patients with histologic grade III tumors than in tissues of other patients (15.00 % *vs.* 57.30 % *vs.* 75.00 % for grades I, II and III, respectively; *P* = 0.001). Furthermore, POU5F1/Oct-4 expression levels were higher in molecular subtype HER2+ than in luminal A, luminal B and basal-like subtypes (83.33 % *vs.* 63.64 % *vs.* 52.00 % *vs.*36.95 %; *P* = 0.030). When Oct-4 was overexpressed in a tissue, HER2 was also overexpressed in the same tissue (Fig. 2). The expression rate of POU5F1/Oct-4 was lower in DCIS than in mucinous carcinoma, IDC and Paget’s disease; but the difference was not statistically significant (40.00 % *vs.* 44.44 % *vs.* 57.65 % *vs.* 100.00 %; *P* = 0.216). The difference in POU5F1/Oct-4 expression level between pT1 (including Tis) tumors and pT2 and pT3 tumors was not statistically significant (52.05 % *vs.* 51.11 % *vs.* 66.67 % for pT1 *vs.* pT2 *vs.* pT3, respectively; *P* = 0.873); and the difference in POU5F1/Oct-4 expression level among patients in different age groups was also not statistically significant (*P* = 0.346). Details are presented in Table 2. Representative images of POU5F1/Oct-4 staining are shown in Fig. 3.

**Table 2:** POU5F1/Oct-4 expression in groups classified by different characteristics

Variables	Number	Oct-4-	Oct-4+	*P*
Age				0.579
<40	26	12	14	
40–60	72	37	35	
>60	23	9	14	
Tumor size				0.873
T1 (Tis)	73	35	38	
T2	45	22	23	
T3	3	1	2	
Histological grade				0.001**
I	20	17	3	
II	89	38	51	
III	12	3	9	
Pathology classification				0.216
DCIS	25	15	10	
IDS	85	36	49	
Mucinous carcinoma	9	5	4	
Paget’s disease	2	0	2	
Molecular classification				0.030*
Luminal A	44	16	28	
Luminal B	25	12	13	
Basal-like	46	29	17	
HER-3+	6	1	5	
Lymph node metastasis				0.003*
pN_0_	83	48	35	
pN_1_	33	10	23	
pN_2_	5	0	5	

*P < 0.05; **P < 0.01

### POU5F1/Oct-4 expression and non-SLN metastasis

Among the 121 patients enrolled in this study, 38 patients had SLN metastasis. Among these 38 patients, 23 patients had non-SLN metastasis and 15 patients were free from non-SLN metastasis. Furthermore, among these 38 patients, 28 patients were POU5F1/Oct-4 positive. Through follow-ups and pathologic examinations, most SLN-metastasis-free patients were found to be free from non-SLN metastasis. The expression rate of POU5F1/Oct-4 was significantly higher in patients with non-SLN metastasis than in patients free from non-SLN metastasis (87 % *vs.* 53.3 %, *P* = 0.03).

### POU5F1/Oct-4 expression and immunohistochemical markers

Chi-square analysis was performed to investigate the relationship between POU5F1/Oct-4 expression and immunohistochemical markers. Results revealed that none of the markers were related to POU5F1/Oct-4 expression (Table 3).

**Table 3:** Association between POU5F1/Oct-4 expression and common factors

Variables	Number	Oct-4^−^	Oct-4^+^	*P*
EG receptor				0.954
Negative	56	27	29	
Positive	65	31	34	
PG receptor				0.479
Negative	69	35	34	
Positive	52	23	29	
HER2/nue				0.745
Score 0+, 1+, 2+	111	57	54	
Score 3+	10	6	4	
P53				0.059
Negative	85	36	49	
Positive	36	22	14	

### Validation of the MSKCC nomogram

**Fig. 4:**
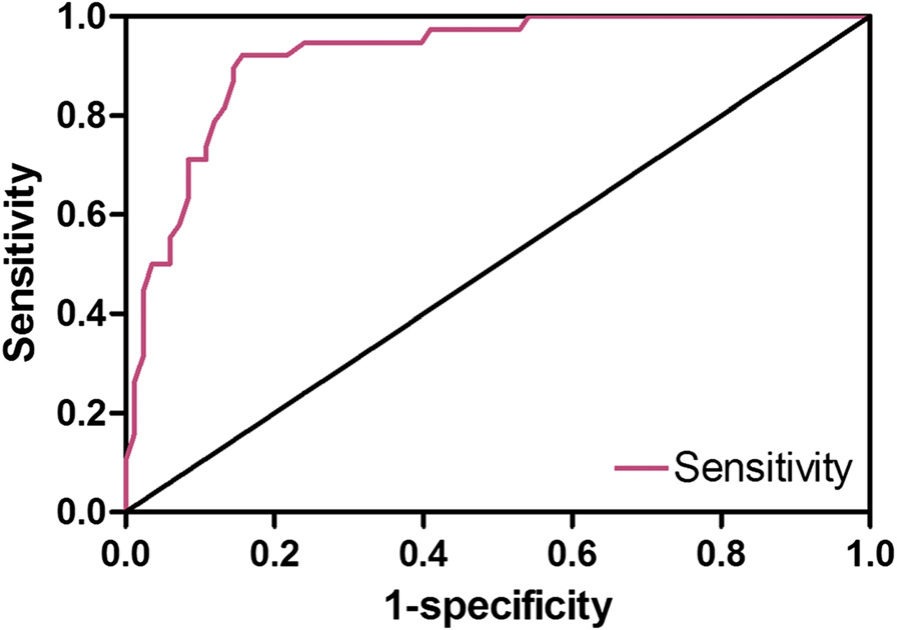
ROC of the MSKCC nomogram in predicting SLN metastasis

The relationship between POU5F1/Oct-4 expression levels and the MSKCC nomogram is illustrated in Fig. 1. The X-axis represents the possibility of non-SLN metastasis generated by the MSKCC nomogram, and the Y-axis represents the proportion of samples that POU5F1/Oct-4 expressed. Although the actual metastasis rate reached 100 % when the probability of the MSKCC nomogram almost reached 70 %, it can be obviously observed that the probability generated by the MSKCC nomogram increased as the actual metastasis rate increased, as shown in Fig. 1. This indicates that the nomogram may also be valid in the population of Liaoning Province. The ROC based on MSKCC nomogram results is shown in Fig. 4. It can be clearly observed that the nomogram has high sensitivity. The AUC of the ROC is 0.919 (95 % CI: 0.869-0.969, *P* < 0.001), which indicates that the MSKCC nomogram may excellently detect the metastasis of SLN in the population of Liaoning Province.

### The relationship between the MSKCC nomogram and POU5F1/Oct-4

**Fig. 5:**
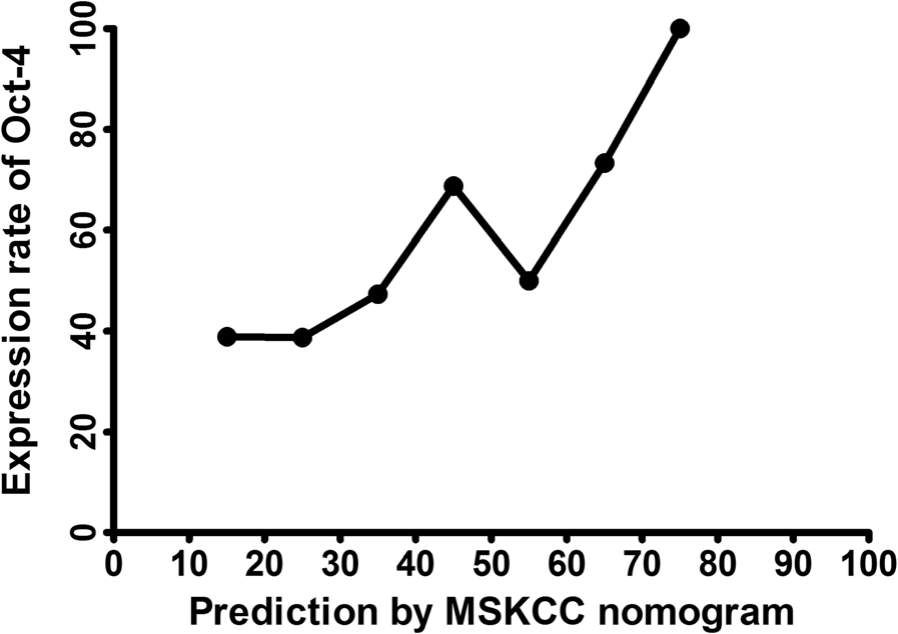
The proportion of samples with high expression levels of POU5F1/Oct-4 *vs.* the MSKCC nomogram’s probability of SLN metastases is shown

Generally, POU5F/Oct-41 expression level increases when the probability of SLN metastasis generated from the MSKCC nomogram increases, as shown in Fig. 5. For non-SLN metastases, both univariate and multivariate analysis results revealed that POU5F1/Oct-4 was significantly associated with non-SLN metastases (*P* = 0.029 and *P* = 0.034, respectively). This might indicate that Oct-4 has the potential to be a predictor of non-SLN metastases. Multivariate analysis results are shown in Table 4.

**Table 4:** Multivariate analysis results on the association between Oct-4 expression and non-SLN metastases

Variables	OR	95 % CI	*P*
Age	1.032	0.964–1.105	0.359
ER	0.512	0.035–7.411	0.623
PR	0.397	0.061–2.606	0.336
CerbB2	0.775	0.141–4.258	0.770
P53	2.893	0.546–15.32	0.212
Oct-4	7.880	1.164–53.344	0.034*

*OR* Odds Ratio; *P < 0.05

## Discussion

POU5F1/Oct-4 is a member of the Pit-Oct-Unc (POU) transcription factor family [19] and is highly expressed initially in the early stages of embryogenesis. With the development of the embryo, its function in cells is restricted in the inner cell mass [20]. POU5F1/Oct-4 can keep embryonic stem cells undifferentiated. Although the precise function of POU5F1/Oct-4 and how its expression regulate the differentiation of ESCs remains unelucidated, it has been considered to play a key role in regulating the identity of ESCs. To date, studies have revealed that POU5F1/Oct-4 can maintain the pluripotency of cells by restricting some important developmental transcription factors such as Pax6, Otx1, and HoxB1 [21]. However, there are currently few studies on POU5F1/Oct-4 as an indicator of lymph node metastasis.

Previous studies have already identified several eligible indicators that could help patients keep free from a complete axilliary dissection. However, evaluating non-SLN metastases with a single indicator seems more or less arbitrary; thus, evaluating more indicators should be recommended at present. This study is the first to determine that positive POU5F1/Oct-4 expression levels are associated with SLN metastases and non-SLN metastases in early-stage breast cancer patients. We also found that the expression of POU5F1/Oct-4 was related to both histological and molecular type. This implies that the status of the expression of POU5F1/Oct-4 in tumor tissues has the potential to act as a predictor for SLN metastases. Moreover, multivariate analysis results revealed that POU5F1/Oct-4 was significantly associated with non-SLN metastases, which indicates that high POU5F1/Oct-4 expression levels may be an indicator of non-SLN metastasis in breast cancer patients. This findings can be useful as a reference to help doctors in diagnosing patients, which may exempt many patients from undergoing complete auxiliary dissection; thus, improving the quality of life of breast cancer patients.

There are some flaws in the validation of the MSKCC nomogram. First,the sample size in this study was relatively small compared to previous studies [22–24]; therefore, random errors may exist. Second, the nomogram itself may not perfectly perform in patients from Liaoning Province of China, since the nomogram was built based on the population of patients in the United States. Therefore, confounders such as ethnic or environmental factors may account for this. There is a need for further studies that aim to validate the nomogram for patients in China.

The Department of Surgical Oncology of our hospital is affiliated with one of the largest hospitals (The First Affiliated Hospital of China Medical University) in Shenyang and in Northeast China. Therefore, this department is not only a local institution in Shenyang city, but also a regional center for patients from Northeast China; which is composed of three provinces and has a total population of over 100 million. Since the validation of the MSKCC nomogram seems to be excellent, we can infer that the nomogram may also be valid for the population in Northeast China.

However, there are some limitations in our study. First, the retrospective nature of this study and its population-based design might induce selection bias. Compared to previous studies, the sample size was not large enough to present a strongly confirmed relationship between expression of POU5F1/Oct-4 and non-SLN metastasis. Second, the validation of the MSKCC nomogram appears not completely convincible due to the lack of patients with a predicted probability above 80 %. In this study, only three patients had a predicted probability above 70 %. More studies that could demonstrate the newly discovered association between the expression of POU5F1/Oct-4 and non-SLN metastasis are needed, in order to explore other new predictors that might exempt breast cancer patients from undergoing complete axillary dissection. Moreover, more data from breast cancer patients of other populations needs to be collected to validate and calibrate the MSKCC nomogram; which can help improve its performance for each population.

## Conclusions

POU5F1/Oct-4 expression levels in breast cancer tissues are significantly associated with SLN and non-SLN metastasis, indicating that POU5F1/Oct-4 is a potential candidate for predicting metastasis. The MSKCC nomogram may perform well with the population in Northeast China, and the predication of metastasis by POU5F1/Oct-4 also accords with the results of the MSKCC nomogram. More studies on POU5F1/Oct-4 are needed to further explore its potential of being a predictor for SLN and non-SLN metastasis in breast cancer tissue.

## Abbreviations

non-SLN: non-sentinel lymph node
MSKCC: Memorial Sloan-Kettering Cancer Center
ROC: receiver operating characteristic
AUC: area under the curve
DAB: diaminobenzidine
DCIS: ductal carcinoma *in situ*
